# Attempted Restoration of Elbow Flexion in Suspected Parsonage-Turner Syndrome Using the Oberlin Procedure: A Case Report With Pending Outcomes

**DOI:** 10.7759/cureus.112916

**Published:** 2026-07-18

**Authors:** Quinn M Jackson, Stephen S Burks

**Affiliations:** 1 Medicine, Dr. Kiran C. Patel College of Osteopathic Medicine, Nova Southeastern University, Fort Lauderdale, USA; 2 Neurosurgery, University of Miami Miller School of Medicine, Miami, USA

**Keywords:** brachial plexopathy, double oberlin nerve transfer, musculocutaneous nerves, nerve surgery, oberlin procedure, parsonage-turner syndrome (pts), peripheral nerve injury (pni), peripheral nerves, ulnar nerve surgery, ulnar nerve (un)

## Abstract

Parsonage-Turner syndrome (PTS) can present with upper extremity weakness that overlaps clinically with traumatic upper-trunk brachial plexus injury, cervical radiculopathy, and shoulder pathology. Although many patients improve with conservative management, persistent paralysis of elbow flexion after a defined period of observation creates a difficult management decision. In selected patients with preserved donor nerve function and no evidence of meaningful reinnervation, distal nerve transfer may be considered to attempt restoration of elbow flexion.

A 58-year-old right-hand-dominant man developed progressive right upper extremity weakness after a skiing accident in which he fell onto his right shoulder and was initially diagnosed with a rotator cuff tear. He later sustained a second fall onto his left palm. The differential diagnosis included traumatic upper-trunk brachial plexus injury, cervical radiculopathy related to prior cervical surgery, rotator cuff pathology, recurrent tendon injury, and PTS or idiopathic brachial neuritis. Suspicion for PTS was supported by delayed progression of weakness after the inciting event, absence of a compressive cervical lesion, electrodiagnostic denervation in the musculocutaneous distribution, and preserved median and ulnar donor function. Although he reported dropping objects and difficulty using the right hand during daily tasks, formal distal motor testing showed preserved finger flexion and abduction. After six months of observation and therapy without recovery of antigravity elbow flexion, surgical exploration was performed. A double fascicular transfer was completed using the flexor carpi radialis fascicle of the median nerve to the biceps branch of the musculocutaneous nerve and the flexor carpi ulnaris fascicle of the ulnar nerve to the brachialis branch of the musculocutaneous nerve. The immediate postoperative course was stable with preserved donor motor function. Follow-up is not yet available.

This case describes the attempted restoration of elbow flexion in a suspected non-recovering PTS using a double fascicular transfer. Because long-term functional outcome data are pending, the report emphasizes diagnostic uncertainty, careful documentation of failed recovery, preservation of donor function, and the need to balance continued observation against the risk of delayed reinnervation.

## Introduction

Parsonage-Turner syndrome (PTS), also known as neuralgic amyotrophy, is a peripheral neuropathy that continues to challenge both neurologists and neurosurgeons because of its unpredictable course and wide spectrum of outcomes. It often begins suddenly after viral illness, vaccination, or trauma, with severe shoulder or arm pain followed by profound weakness and wasting of the affected muscles. Although once considered self-limiting, recent studies show that many patients develop chronic pain, fatigue, and disabling weakness that persist despite conservative management [1]. The most commonly affected muscles include the deltoid, supraspinatus, biceps, and serratus anterior, with elbow flexion frequently compromised when the upper-trunk or musculocutaneous distribution is involved [1].

The natural history of recovery is highly variable. Some patients regain functional strength within one to two years, while others have persistent weakness, incomplete recovery, or limited antigravity function [2]. Electromyography (EMG) may reveal early denervation and, in severe cases, an absence of reinnervation potentials even after prolonged follow-up. Modern imaging techniques, including magnetic resonance neurography and ultrasound, have demonstrated focal constrictions and fascicular distortions known as hourglass lesions, reframing the syndrome as one that can involve structural pathology rather than purely transient inflammation [3].

Conservative care remains appropriate for those who improve, but patients without recovery face permanent disability. Loss of elbow flexion reduces independence with difficulty in feeding and grooming, and continued denervation eventually leads to irreversible motor endplate loss and muscle fibrosis [1,2]. Experimental and clinical data confirm that delayed reinnervation significantly reduces functional recovery, supporting the principle of timely surgical intervention [4].

Distal nerve transfers have become a mainstay of neurosurgical reconstruction for upper-trunk and musculocutaneous deficits. By transferring expendable fascicles from the median or ulnar nerve to musculocutaneous branches, surgeons shorten the regeneration distance and improve functional outcomes. Although first developed for traumatic brachial plexus injuries, their application has expanded to non-traumatic and idiopathic causes such as PTS when recovery fails [5,6]. Double fascicular transfer, in which both the biceps and brachialis are reinnervated, provides greater torque and endurance than single fascicular transfer and has shown excellent outcomes in both early and delayed reconstructions [7,8].

This case describes a 58-year-old man with suspected PTS who failed to recover after six months of observation and structured therapy. Persistent paralysis of elbow flexion with preserved donor nerves created an opportunity for surgical reconstruction. The decision to proceed with double fascicular transfer highlights the evolving role of neurosurgeons in managing patients whose weakness does not resolve spontaneously.

## Case presentation

A 58-year-old right-hand-dominant man presented with disabling weakness of the right upper extremity after a reported fall while skiing six months prior. He reported falling onto his right shoulder and was diagnosed with a rotator cuff tear. Shortly after, he sustained a second fall onto his left palm. The patient reported no immediate weakness following the fall but developed extremity weakness over the subsequent months. He reported no pain at the time of the visit but reported difficulty with pronation and flexion of the right upper extremity. He denied numbness or tingling in the right upper extremity. From the time of the accident, the patient reported difficulty using the right upper extremity during daily activities, including clumsiness, difficulty writing, and dropping objects. However, formal distal motor testing later demonstrated preserved finger flexion and abduction, suggesting that these functional complaints were likely related to impaired arm positioning and loss of elbow flexion rather than intrinsic hand paralysis.

Following the initial inciting event, he completed a course of physical therapy without reported improvement. He had a recovery of shoulder function without any improvement in elbow flexion or supination. The inability to adequately feed himself, brush his teeth, button his clothing, write, or lift even light objects prompted referral for surgical evaluation. EMG obtained early confirmed denervation in the musculocutaneous distribution without reinnervation potentials. Cervical MRI demonstrated postoperative changes related to prior fusion without recurrent compression, foraminal stenosis, or mass lesion to explain the deficit. Brachial plexus MRI neurography and ultrasound were not obtained before surgery. Therefore, no radiographic confirmation of hourglass-like constriction or focal fascicular abnormality was available in this patient.

Past surgical history included anterior cervical discectomy and fusion at C5-C7 and subsequent lumbar decompression with resolution of prior radicular symptoms. Additional history included a remote biceps tendon tear, kidney stone removal, and colon surgery. Although his past medical history raised concern for a possible cause of his right upper extremity weakness, clinical and electrodiagnostic evaluation raised concern for PTS or idiopathic brachial neuritis.

On physical examination, he appeared in no distress and was fully oriented. The right upper limb showed hollowing of the anterior arm with visible atrophy of the biceps and brachialis. He could not lift the forearm from the table when asked to flex the elbow. Supination was absent. Shoulder abduction was preserved against mild resistance. Hand strength was normal, with intact finger flexion and abduction. Sensation was grossly intact across dermatomes with a subtle reduction along the lateral forearm. Deep tendon reflexes were symmetric except for an absent right biceps reflex. The pertinent physical exam findings can be seen in Tables 1-3.

**Table 1:** Motor Examination Manual muscle testing of the upper extremities using the Medical Research Council scale.

Muscle group	Right-sided strength grade	Left-sided strength grade
Shoulder abductors	4/5	5/5
Elbow flexors	1/5	5/5
Wrist extensors	5/5	5/5
Elbow extensors	5/5	5/5
Finger flexors	5/5	5/5
Finger abductors	5/5	5/5

**Table 2:** Sensory Examination Sensory examination findings of the upper extremities.

Modality	Right upper extremity	Left upper extremity
Light touch	Subtle decrease over lateral forearm	Normal
Vibration	Normal	Normal
Pin prick	Normal	Normal
Proprioception	Normal	Normal

**Table 3:** Reflex Examination Deep tendon reflexes were graded bilaterally, highlighting an absent right biceps reflex with otherwise symmetric responses.

	Biceps reflex	Triceps reflex	Brachioradialis reflex
Right extremity	0/4	2/4	2/4
Left extremity	2/4	2/4	2/4

An EMG was obtained during the patient’s initial workup and was documented as showing denervation in the musculocutaneous distribution without evidence of reinnervation. This supported involvement of the biceps and brachialis muscles and contributed to concern for a non-recovering upper-trunk or musculocutaneous process. Median and ulnar motor function was preserved clinically and intraoperatively, supporting their use as donor nerves for transfer. The full EMG report was not available for review in the documentation used for this case report; therefore, the specific muscles tested, presence or absence of fibrillation potentials or positive sharp waves, motor unit morphology, and recruitment patterns could not be independently confirmed. Serial EMG was also not available.

The presentation reflected a profound right upper extremity deficit consistent with chronic upper-trunk palsy. Although PTS remained a leading diagnosis, the differential diagnosis also included traumatic upper-trunk brachial plexus injury, cervical radiculopathy, rotator cuff pathology, and recurrent tendon injury. Because recovery in PTS can continue over 12 to 24 months, continued observation was considered. However, this patient had persistent absence of antigravity elbow flexion after six months, severe functional impairment, documented EMG evidence of denervation without reported reinnervation, and preserved median and ulnar donor function. Surgical exploration with possible nerve transfer was therefore recommended after weighing the possibility of late spontaneous recovery against the risk that further delay could reduce the likelihood of successful reinnervation.

Procedure description

The patient was brought to the operating room, intubated, and positioned supine with the right arm on an arm board. The extremity was prepped and draped in sterile fashion. A longitudinal skin incision was made along the bicipital groove and carried distally along the humerus. Dissection proceeded through the subcutaneous tissue and fascia to the interval between the biceps and brachialis. The musculocutaneous nerve was identified deep to the biceps muscle. Its branches to the biceps and brachialis muscles, as well as the lateral antebrachial cutaneous nerve, were skeletonized under loupe magnification. Intraoperative stimulation at 10 mA produced no or only minimal contraction in the target muscles, consistent with denervation.

For the biceps transfer, the musculocutaneous branch to the biceps was divided proximally and prepared for repair. The median nerve was exposed adjacent to the brachial artery and mobilized. Internal neurolysis was performed under the operating microscope to separate discrete fascicular groups. A donor fascicle to the flexor carpi radialis was identified with stimulation and divided distally to preserve donor function. The recipient and donor ends were brought into gentle apposition without tension, with the elbow flexed. An epineurial coaptation was performed using interrupted 8-0 nylon sutures under high magnification. The neurorrhaphy was reinforced with fibrin sealant and wrapped with a sleeve of porcine submucosa to provide protection and reduce perineural scarring.

**Figure 1:**
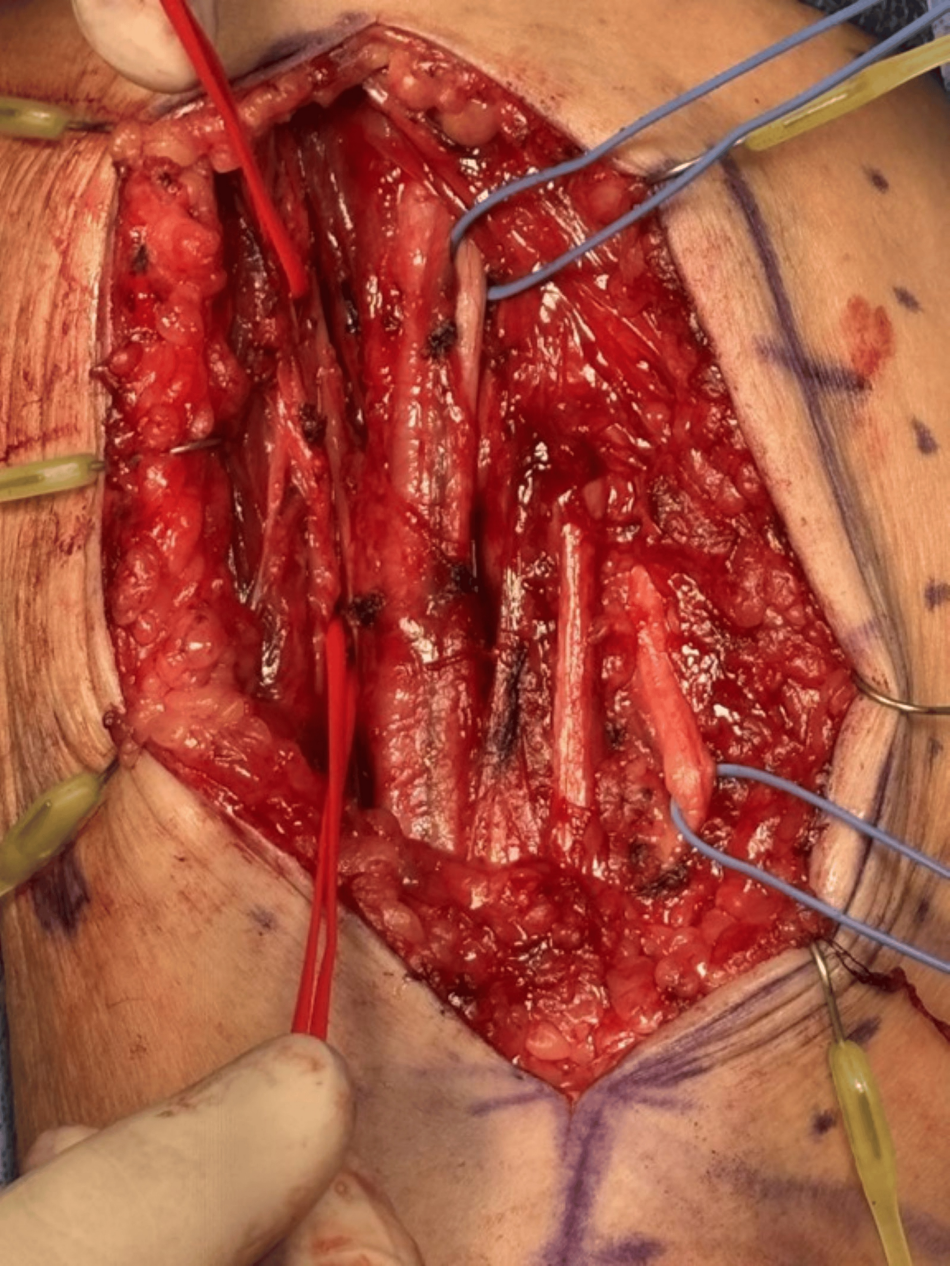
Intraoperative View of the Right Upper Extremity During the Oberlin Procedure Intraoperative view demonstrating the musculocutaneous nerve branches to the biceps and brachialis muscles, the lateral antebrachial cutaneous nerve, the median nerve, the ulnar nerve, and the brachial artery and vein. Red vessel loops mark the proximal biceps branch and distal brachialis branch of the musculocutaneous nerve.

For the brachialis transfer, the musculocutaneous branch to the brachialis was divided proximally and prepared. The ulnar nerve was exposed and underwent careful internal neurolysis under the microscope. A donor fascicle to the flexor carpi ulnaris was identified with stimulation and divided distally. The fascicle was coapted to the brachialis branch with interrupted 8-0 nylon epineurial sutures in a tension-free fashion. Fibrin sealant was applied, and the repair was circumferentially wrapped with porcine submucosa that was anchored proximally and distally to function as a biologic conduit. Pertinent neuroanatomy and surgical landmarks are shown in Figure 1.

Both coaptation sites were inspected to confirm stable alignment, absence of rotation, and a dry field. Hemostasis was achieved with copious irrigation and bipolar coagulation as needed. The wound was closed in layers with absorbable sutures, and a sterile dressing was applied. Estimated blood loss was less than 50 mL. There were no intraoperative complications. In the immediate postoperative period, the hand was warm and well perfused with intact capillary refill. Donor motor function in the forearm and hand was preserved on exam. The right upper extremity was immobilized postoperatively to protect the coaptation sites; the exact immobilization angle was not available in the operative documentation. Rehabilitation was planned to begin after immobilization, with early synergy training followed by progressive cortical retraining and strengthening once reinnervation is observed. Outpatient follow-up is not yet available.

## Discussion

This case illustrates the difficult balance between observation and intervention in PTS. The delayed progression of elbow flexion loss, absence of ongoing pain at surgical evaluation, and preserved donor function supported an idiopathic neuropathy rather than an immediate direct traumatic palsy. After six months of observation and therapy, there was no return of function, and EMG continued to show denervation without reinnervation, creating a poor prognosis for spontaneous recovery and justifying surgery. Proceeding with reconstruction at this stage was consistent with evidence that prolonged denervation can reduce the likelihood of meaningful recovery [4,5].

It is important to emphasize that surgery is not appropriate for most patients with PTS. Many patients improve with observation, pain control, and therapy, and recovery may continue for 12 to 24 months. Operative treatment should therefore be reserved for carefully selected patients with persistent disabling weakness, electrodiagnostic evidence of denervation without meaningful reinnervation, and preserved donor nerves. The risks of nerve transfer include donor fascicle morbidity, failed reinnervation, incomplete cortical retraining, and persistent weakness despite technically successful coaptation. In this case, surgery was considered because the patient had no return of antigravity elbow flexion after six months and remained severely limited in activities of daily living.

The timing of surgery is one of the central issues in this case. Many patients with PTS improve without surgery, and recovery may continue for 12 to 24 months. For that reason, nerve transfer should not be considered routine at six months. In this patient, the decision was individualized. He had a persistent inability to flex the elbow against gravity, severe loss of independence in daily activities, and electrodiagnostic denervation without documented reinnervation. Waiting longer may have allowed late spontaneous recovery, but it also risked progressive loss of motor endplate viability and a narrower reconstructive window. The decision to proceed was therefore based on the severity and persistence of the deficit rather than the diagnosis of PTS alone.

Double fascicular transfer was chosen to maximize the chance of restoring elbow flexion. In this patient, donor integrity was preserved, with intact median and ulnar function. This allowed selective harvest of expendable fascicles to reinnervate both biceps and brachialis, ensuring greater torque and endurance than would be expected with a single fascicle transfer. Recent neurosurgical series confirm the reliability of this approach in both traumatic and idiopathic palsies, showing consistent antigravity recovery and preservation of donor hand strength [7].

The procedure was completed without intraoperative complications, and immediate postoperative recovery was stable, without wound issues, sensory loss, new motor deficit, or loss of donor function. These details support the conclusion that the transfer was technically successful and safe. Rehabilitation was planned to begin with synergy training and progress to cortical retraining, reflecting current best practice after nerve transfer and addressing the need to translate axonal regeneration into function [8].

Although the onset of weakness followed a reported accident, the delayed and painless progression suggested an etiology distinct from an immediate direct traumatic palsy. Literature emphasizes that PTS can develop after minor trauma, vaccination, or systemic illness, reflecting an immune-mediated inflammatory neuropathy rather than a purely mechanical insult [1,2]. High-resolution magnetic resonance neurography has demonstrated hourglass-like fascicular constrictions in some EMG-confirmed cases of neuralgic amyotrophy [3]. However, these structural findings were not confirmed in this patient because brachial plexus MRI neurography and ultrasound were not obtained. In this case, suspected PTS was based on the clinical timeline, absence of compressive cervical pathology, and documented electrodiagnostic denervation rather than radiographic confirmation of fascicular constriction.

Recognizing this distinction is clinically important, as the prognosis and management strategy differ substantially. Traumatic lesions may warrant early exploration, whereas idiopathic neuritis is initially treated conservatively with observation and therapy. When functional recovery fails after a defined period, timely referral for neurosurgical evaluation becomes critical. Prolonged denervation beyond 12 months has been shown to reduce the likelihood of successful reinnervation [4]. Recent case series emphasize that failure to recognize PTS as a separate entity can delay electrodiagnostic confirmation and definitive reconstruction [5,9]. In this patient, the absence of recovery after six months justified surgical nerve transfer, aligning with current evidence supporting intervention once continued denervation and poor reinnervation potential are confirmed.

This case also demonstrates the evolving surgical role in selected non-recovering cases of suspected PTS. While imaging-confirmed hourglass constrictions were not demonstrated in this patient, recent literature has expanded the understanding of PTS from a purely medical condition to one that may, in selected cases, require peripheral nerve surgical evaluation [5,9]. For neurosurgeons, the relevance of this case is not that PTS should routinely undergo surgery, but that persistent disabling weakness without recovery may warrant timely referral and individualized discussion of reconstructive options.

The main limitation is the absence of longitudinal follow-up, which prevents reporting of time to first contraction, strength milestones, dynamometry, range of motion, and patient-reported outcomes. Diagnostic certainty is also limited by the lack of confirmatory brachial plexus MRI neurography or ultrasound. Although recent literature has described hourglass-like constrictions and focal fascicular abnormalities in PTS, these findings were not confirmed in this patient. A further limitation is that the EMG was documented as showing denervation without reinnervation, but the full electrodiagnostic report was not available for review. As a result, the specific muscles tested, spontaneous activity, motor unit morphology, recruitment patterns, and serial electrodiagnostic progression could not be reported. Finally, the effect of rehabilitation intensity and the durability of donor preservation can only be assessed with time.

Because outpatient follow-up is not yet available, this case cannot establish the effectiveness of the transfer or confirm functional recovery. Rather, it illustrates the decision-making process for the attempted restoration of elbow flexion in suspected non-recovering PTS.

## Conclusions

This case describes the attempted restoration of elbow flexion in a patient with suspected non-recovering PTS after six months of observation and therapy. Preserved median and ulnar donor function, persistent absence of antigravity elbow flexion, and documented electrodiagnostic denervation without reported reinnervation supported surgical exploration and double fascicular transfer. Because outpatient follow-up is not yet available, no conclusion can be drawn regarding long-term functional recovery. This report instead highlights the importance of careful diagnostic evaluation, transparent discussion of uncertainty, and longitudinal reporting after nerve transfer in suspected non-recovering PTS.
